# The use of sodium-glucose co-transporter-2 inhibitors or glucagon-like peptide-1 receptor agonists versus sulfonylureas and the risk of lower limb amputations: a nation-wide cohort study

**DOI:** 10.1186/s12933-023-01897-2

**Published:** 2023-06-29

**Authors:** Nikki C. C. Werkman, Johanna H. M. Driessen, Coen D. A. Stehouwer, Peter Vestergaard, Nicolaas C. Schaper, Joop P. van den Bergh, Johannes T. H. Nielen

**Affiliations:** 1 Cardiovascular Research Institute Maastricht (CARIM), Maastricht University, Maastricht, The Netherlands; 2 Department of Clinical Pharmacy and Toxicology, Maastricht University Medical Center+, Maastricht, The Netherlands; 3 School for Nutrition and Translational Research in Metabolism (NUTRIM), Maastricht University, Maastricht, The Netherlands; 4 Department of Internal Medicine, Maastricht University Medical Center+, Maastricht, The Netherlands; 5 Department of Clinical Medicine, Aalborg University, Aalborg, Denmark; 6 Steno Diabetes Center North Denmark, Aalborg University Hospital, Aalborg, Denmark; 7 Department of Endocrinology, Aalborg University Hospital, Aalborg, Denmark; 8 Department of Internal Medicine, Division of Endocrinology, Maastricht University Medical Center+, Maastricht, The Netherlands; 9 Department of Internal Medicine, Division of Rheumatology, Maastricht University Medical Center+, Maastricht, The Netherlands; 10 Department of Internal Medicine, VieCuri Medical Center, Venlo, The Netherlands

**Keywords:** Type 2 diabetes, Sodium-glucose co-transporter-2 inhibitors, Glucagon-like peptide-1 receptor agonists, Amputations, Diabetic foot ulcer, Cohort study

## Abstract

**Background:**

Numerous studies have investigated the potential association of sodium-glucose co-transporter-2 inhibitors (SGLT2-Is) with an increased risk of lower limb amputations (LLAs), but have produced conflicting results. Particularly studies comparing SGLT2-Is to glucagon-like peptide-1 receptor agonists (GLP1-RAs) seem to find a higher LLA risk with SGLT2-I use. This raises the question whether the results are driven by a protective GLP1-RA-effect rather than a harmful SGLT2-I-effect. GLP1-RAs could promote wound healing and therefore reduce the risk of LLAs, but the associations between both drug classes and LLA remain uncertain. Therefore, the aim of the current study was to investigate the risk of LLA and diabetic foot ulcer (DFU) with SGLT2-I use and GLP1-RA use versus sulfonylurea use.

**Methods:**

A retrospective population-based cohort study was conducted using data from the Danish National Health Service (2013–2018). The study population (N = 74,475) consisted of type 2 diabetes patients aged 18 + who received a first ever prescription of an SGLT2-I, GLP1-RA or sulfonylurea. The date of the first prescription defined the start of follow-up. Time-varying Cox proportional hazards models estimated the hazard ratios (HRs) of LLA and DFU with current SGLT2-I use and GLP1-RA use versus current SU use. The models were adjusted for age, sex, socio-economic variables, comorbidities and concomitant drug use.

**Results:**

Current SGLT2-I use was not associated with a higher risk of LLA versus sulfonylureas {adjusted HR 1.10 [95% confidence interval (CI) 0.71–1.70]}. Current GLP1-RA use, on the other hand, was associated with a lower risk of LLA [adjusted HR 0.57 (95%CI 0.39–0.84)] compared to sulfonylureas. The risk of DFU was similar to that with sulfonylureas with both exposures of interest.

**Conclusion:**

SGLT2-I use was not associated with a higher risk of LLA, but GLP1-RAs with a lower risk of LLA. Previous studies reporting a higher risk of LLA with SGLT2-I use compared to GLP1-RA use might have been looking at a protective GLP1-RA effect, rather than a harmful SGLT2-I effect.

**Supplementary Information:**

The online version contains supplementary material available at 10.1186/s12933-023-01897-2.

## Background

Lower limb amputation (LLA) is a highly feared outcome of type 2 diabetes (T2D) [1]. LLAs not only have a profound impact on the patient’s life, but are also associated with great economic burden [2] and high mortality rates [3]. LLAs are approximately ten times more common among individuals with diabetes compared to those without [4]. Particularly at risk of amputation are patients with diabetic foot ulcer (DFU) and peripheral arterial disease (PAD) [5].

Sodium-glucose co-transporter-2-inhibitors (SGLT2-Is) form a new class of drugs used in treating T2D. These drugs exert their glucose-lowering effect by preventing the reabsorption of plasma glucose and sodium in the proximal renal tubule [6]. The use of SGLT2-Is has been associated with lower mortality as well as positive cardiac and renal outcomes [7–9]. However, they have also been associated with several safety concerns, such as a higher risk of LLA compared to placebo [10].

The first report of a higher risk of LLA associated with the use of the SGLT2-I canagliflozin in the CANagliflozin CardioVascular Assessment Study (CANVAS) Program [10] led to substantial concern. Subsequently, numerous studies were performed to investigate this potential association. Individual observational studies produced conflicting results, whereas meta-analyses reported no higher risk of LLA associated with SGLT2-I use [9, 11]. The wide range of contrasting findings may have resulted from differences in study population and study design, especially the use of different reference groups. Interestingly, two studies in which SGLT2-Is were compared to glucagon-like peptide-1 receptor agonists (GLP1-RAs) (another new class of glucose-lowering drugs) both reported a higher risk of LLA associated with SGLT2-I use [12, 13]. These findings raise the question whether the results of these studies could have been driven by a GLP1-RA-effect rather than an SGLT2-I-effect. Indeed, the GLP1-RA liraglutide has tentatively been associated with a lower risk of LLA compared to placebo [14]. The use of the GLP1-RA exenatide, however, was not associated with LLA risk versus placebo [15]. As a result, the association between SGLT2-I use and LLA remains uncertain.

SGLT2-I induced hypovolemia with reduced tissue perfusion has been proposed as a potential underlying pathway leading to LLA [16], but this mechanism has not been confirmed. A previous study reported no association between SGLT2-I use and LLA, irrespective of the presence of signs hypovolemia [17]. However, this study was limited by a low number of events, possibly because of the use of a primary care database in which LLAs might have been under recorded. LLA registration is likely to be more accurate in hospital registries using amputation procedure codes, such as the Danish National Patient registry [18].

Ultimately, the presence of an association between SGLT2-Is and LLA, the potential role of hypovolemia, and the influence of the use GLP1-RAs as a reference group remain unclear. Therefore, the primary aim of the current study was to investigate the risk of LLA with the use of SGLT2-Is or GLP1-RAs versus other antidiabetic drugs, using the Danish National Patient registry. The secondary aim was to study whether the presence of signs of hypovolemia or PAD is associated with a higher risk of LLA among SGLT2-I users, in order to assess whether these characteristics could be involved in the underlying pathway.

## Methods

We conducted a retrospective cohort study using data from the Danish National Health Service. The Danish Civil Registry System assigns a unique 10-digit civil registry (CPR) number to all Danish residents at birth or immigration. This number is consistently used across all Danish registries and allows for linkage between the population-based registries [19]. Data on changes in vital status, including date of death and changes of address are registered in the Civil Registration System since 1968. Data on hospital admissions have been recorded since 1977 in the Danish National Hospital Registry. This registry covers 99.4% of all discharge records from Danish hospitals and includes all inpatient contacts, outpatient visits to the hospital, and outpatient visits to clinics and emergency rooms [20, 21]. Diagnoses are registered according to the Danish version of the International Classification of Diseases 10th revision (ICD-10) since 1994. Surgical procedures are registered according to the Danish version of the Nordic Medico-Statistical Committee Classification of Surgical Procedures since 1996. Data on refundable drug prescriptions have been recorded since 1996 in the register of Medicinal Products Statistics of the Danish Medicines Agency [22]. The prescription data provide information on the type and amount of drug prescribed according to the Anatomical Therapeutic Chemical (ATC) classification system, as well as the date on which the prescription was filled.

### Study population

A base cohort of people with diabetes initiating first-line anti-diabetic treatment was created by including all individuals aged 18 years or older with a first metformin prescription between 1997 and 2018. In order to ensure that the individuals had not used any antidiabetic drug before, at least one year of valid data collection prior to the first metformin prescription was required. Patients with a prescription for any antidiabetic drug in the year prior to the first metformin prescription were excluded.

From the base cohort, we selected individuals starting second line treatment with an SGLT2-I, GLP1-RA, sulfonylurea (SU), or dipeptidyl peptidase-4 inhibitor (DPP4-I) (used in a sensitivity analysis) with the first ever prescription between 2013 and 2018. This study period was chosen as SGLT2-Is were introduced in Denmark as of December 2012. The date of the first ever prescription for SGLT2-I, GLP1-RA, SU, or DPP4-I determined the index date. The subsequent processing and analysis of the study cohort is graphically depicted [23] in Additional file 1: Fig. S1.

### Exposure

Drug exposure was determined time dependently by dividing the total duration of follow-up into 30-day intervals. At the start of each interval, exposure to non-insulin glucose lowering drugs (NIGLDs) was determined. Based on the most recent NIGLD prescription, we classified an interval as current (1–90 days) use or past (> 90 days) use. Current use intervals were further stratified into the following mutually exclusive categories of current NIGLD use: SGLT2-I use, GLP1-RA use, SU use, DPP4-I use, combined use (of at least two of the studied drugs), and other NIGLD use (other than the studied drugs). Patients could move between exposure groups during follow-up. The exposures of interest were current SGLT2-I use and current GLP1-RA use, and the reference group was current SU use. SUs were chosen as the reference group since they are used in the same line of treatment of T2D as SGLT2-Is and GLP1-RAs [24], and have no known association with the outcome of interest [25]. The exposures of interest were compared to the reference group in two separate main analyses, and current DPP4-I use was used as a reference group in a sensitivity analysis.

To assess potential mechanisms in the pathways to LLA, current SGLT2-I use and current GLP1-RA use were both further stratified by concomitant use of antihypertensive drugs in the previous 3 months, the presence of signs of hypovolemia in the previous 6 months, and the presence of PAD ever before. Antihypertensive drugs included diuretics and agents acting on the renin–angiotensin–aldosterone system (RAAS). Signs of hypovolemia were identified with ICD-10 codes, including volume depletion (E86), anuria and oliguria (R34), and hypotension (I95). PAD was defined as the presence of a diagnosis based on the ICD-10 code for peripheral vascular disease, unspecified (I73.9) or a history of a peripheral vascularization procedure based on procedure codes.

To assess potential dose- or duration dependent effects, current SGLT2-I use and current GLP1-RA use were both further stratified by cumulative dose and continuous duration of use. The cumulative dose was calculated at each current exposure interval by summing the total amount of previously prescribed study drug (i.e. SGLT2-I or GLP1-RA) in daily defined doses (DDDs) according to the WHO ATC/DDD index [26]. Continuous duration of use was defined as the time from the first prescription until the start of the interval, allowing a gap of 60 days between the estimated end date of a prescription and the start of the next prescription. The estimated end date was based on the number of DDDs provided.

### Outcomes

Individuals were followed from their index date to either the end of data collection, emigration, death, or the outcome of interest, whichever came first. The primary outcome of interest was LLA, defined as any amputation of the femur or below, based on procedure codes NFQ19, NFQ99, NGQ09, NGQ19, NGQ99, NHQ0x, NHQ1x, NHQ99 (with x being: 0 = talocrural; 1 = talocrural and malleoli (Syme); 2 = intertarsal; 3 = tarsometatarsal; 4 = transmetatarsal; 5 = metatarsaophalangeal; 7 = toe, partial). The secondary outcome of interest was forefoot amputation, defined as amputation through or below the metatarsal bones, based on procedure codes NHQ0x and NHQ1x (with x being: 3 = tarsometatarsal; 4 = transmetatarsal; 5 = metatarsaophalangeal; 7 = toe, partial). We were interested in forefoot amputations to gather more information on the potential mechanisms involved; if hypovolaemia plays a role, more distant parts of the lower extremities might be more likely to be affected. DFU was defined using ICD-10 codes for diabetes with foot ulcer (E105B, E115B, E135B, E145B) and open wound on ankle/foot (S91).

### Potential confounders

We identified various risk factors for LLA, which we assessed as potential confounders by reviewing the available data. All potential confounders (with the exception of sex) were determined time-dependently (i.e. at the start of each 30-day interval) and included: sex, age, immigrant status, income category (based on the taxable gross income before deductions in the following categories: low ≤ 30,000 DKK, normal = 30,000–50,000 DKK, high ≥ 50,000 DKK), education category (highest completed education, categorised as basic, secondary, and higher education), duration of T2D (based on the time since first ever metformin prescription), a history of hypertension, ischaemic heart disease, pulmonary heart disease, cerebrovascular disease, PAD, hyperlipidaemia and hypercholesterolaemia, renal disease, retinopathy, neuropathy, bacterial foot infection, fungal foot infection, cellulitis of the lower limb, and osteomyelitis. In addition, the use of the following drugs in the previous 6 months was assessed as potential confounders: insulin, loop diuretics, thiazide diuretics, potassium sparing diuretics, beta-blockers, calcium-channel blockers, digoxin, organic nitrates, antithrombotic agents, lipid-lowering drugs, angiotensin converting enzyme (ACE) inhibitors, and angiotensin II receptor blocker (ARBs). All variables were treated as categorical variables, with the exception of age.

### Statistical analyses

Cox proportional hazards models were used to estimate the hazard ratios (HRs) for the outcomes of interest comparing current SGLT2-I use to current SU use, and current GLP1-RA use to current SU use. Current SGLT2-I use, and current GLP1-RA use were stratified by sex, age, concomitant antihypertensive use (diuretics and drugs acting on RAAS), the presence of signs of hypovolemia, the presence of PAD, cumulative dose and continuous duration of use.

Multivariable analyses were used to address potential confounding in the Cox proportional hazard models. Wald tests were used to compare the results of these stratifications statistically. The HRs were adjusted for age, sex, and the confounders mentioned in the previous section that showed a > 5% change in the beta-coefficient of the sex/age adjusted model. Confounders that must be included as suggested by clinical evidence from literature were included in the model irrespective of the change in beta-coefficient. To avoid overcorrection of the model, we included a maximum of one confounder per 10 events [27]. We calculated the Pearson correlation coefficient between confounders and eliminated or merged any confounders with a correlation coefficient above 0.5. As a sensitivity analysis, we changed the reference group for both main analyses to current DPP4-I use. In a second sensitivity analysis, we excluded individuals with a history of LLA at baseline. In a final sensitivity analysis we evaluated if health care requirement levels influenced the results, by adding the number of concomitant glucose-lowering drugs (categorical: 1 drug, 2–3 drugs, > 3 drugs) and number of bed days (categorical: no days, 1–14 days, 15–29 days, 30–89 days, > 89 days) to the models. Bed days were defined as the total number of days a patient was hospitalised and was based on hospital records. Data was analysed using SAS version 9.4 (SAS Institute, Cary, NC, USA).

## Results

**Fig. 1:**
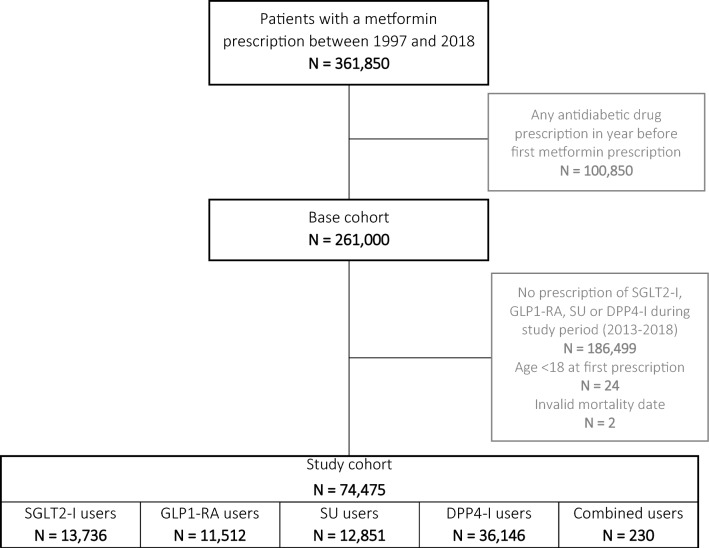
Flow chart showing the selection of patients from the original data extraction, to the base cohort, and to the final study cohort at baseline. Combined users had a prescription of a combination of at least two of the study drugs. *SGLT2-I* sodium-glucose co-transporter-2 inhibitor, *GLP1-RA* glucagon-like peptide-1 receptor agonist, *SU* sulfonylurea, *DPP4-I* dipeptidyl peptidase-4 inhibitor

Figure 1 shows the selection of patients from the original data extraction, yielding a study cohort of 74,475 individuals who started treatment with the exposure(s) of interest. Table 1 shows the baseline characteristics of this study cohort, grouped by drug class of each individual’s index prescription (i.e. the first prescription of a study drug, marking cohort entry). Based on these index prescriptions, 13,736 individuals started treatment with an SGLT2-I, 11,512 with a GLP1-RA, and 12,851 with an SU. The median follow-up time was 1.6 years [inter quartile range (IQR) 0.7–2.9] for SGLT2-I users, 3.8 years (IQR 1.7–5.9) for GLP1-RA users, and 3.8 (IQR 2.1–5.6) for SU users. Median diabetes duration was highest in SGLT2-I users (6.6 years, IQR 2.8–10.1) and lowest in SU users (2.9 years, IQR 0.8–5.6). There were relatively more women among the GLP1-RA users, and their mean age was lower compared to the other groups. Overall, both SGLT2-I users and GLP1-RA users appeared to suffer more from comorbidities (hypertension, hyperlipidaemia, retinopathy, neuropathy) compared to SU users, and insulin use was higher in these two groups. However, renal disease was less common among SGLT2-I users compared to the other groups. There were few recordings of signs of hypovolemia and LLAs in the patients’ history.

**Table 1:** Baseline characteristics of SGLT2-I users, GLP1-RA users, SU users, and DPP4-I users^a,b^

Characteristic	SGLT2-I users N = 13,736	GLP1-RA users N = 11,512	SU users N = 12,851	DPP4-I users N = 36,146
Median follow-up time (years(IQR))	1.6 (0.7–2.9)	3.8 (1.7–5.9)	3.8 (2.1–5.6)	3.1 (1.6–4.9)
Median duration of T2D (years(IQR))	6.6 (2.8–10.1)	5.4 (2.3–8.9)	2.9 (0.8–5.6)	4.1 (1.6–7.1)
Number of women	5283 (38.5)	5183 (45.0)	5379 (41.9)	14,301 (39.6)
Age				
Mean age at index date (years(SD))	59.6 (11.3)	56.3 (12.3)	62.2 (12.8)	62.5 (12.4)
18–49	2467 (18.0)	3203 (27.8)	2123 (16.5)	5529 (15.3)
50–59	4129 (30.1)	3458 (30.0)	3103 (24.1)	8816 (24.4)
60–69	4400 (32.0)	3233 (28.1)	3754 (29.2)	10,786 (29.8)
70 +	2740 (19.9)	1618 (14.1)	3871 (30.1)	11,015 (30.5)
Education^c^				
Basic	4783 (34.8)	4015 (34.9)	5408 (42.1)	13,865 (38.4)
Secondary	6664 (48.5)	5473 (47.5)	5533 (43.1)	16,400 (45.4)
Higher	1893 (13.8)	1718 (14.9)	1411 (11.0)	4713 (13.0)
Missing	396 (2.9)	306 (2.7)	499 (3.9)	1168 (3.2)
Income category				
Low	5312 (38.7)	4674 (40.6)	6717 (52.3)	16,570 (45.8)
Normal	5731 (41.7)	4758 (41.3)	4333 (33.7)	13,406 (37.1)
High	2628 (19.1)	2058 (17.9)	1670 (13.0)	5985 (16.6)
Missing	65 (0.5)	22 (0.2)	131 (1.0)	185 (0.5)
Immigrant status	2585 (18.8)	1868 (16.2)	2591 (20.2)	6404 (17.7)
History of comorbidities				
Hypertension	5288 (38.5)	4422 (38.4)	4228 (32.9)	12,918 (35.7)
Ischaemic heart disease	2872 (20.9)	2252 (19.6)	2496 (19.4)	7227 (20.0)
Pulmonary heart disease	192 (1.4)	202 (1.8)	193 (1.5)	706 (2.0)
Heart failure	945 (6.9)	775 (6.7)	866 (6.7)	2792 (7.7)
Cerebrovascular disease	1158 (8.4)	879 (7.6)	1273 (9.9)	3587 (9.9)
Atherosclerosis	1127 (8.2)	949 (8.2)	1182 (9.2)	3202 (8.9)
Peripheral arterial disease	534 (3.9)	413 (3.6)	552 (4.3)	1714 (4.7)
Hyperlipidaemia	2943 (21.4)	2410 (20.9)	2045 (15.9)	6295 (17.4)
Renal disease	141 (1.0)	222 (1.9)	341 (2.7)	1293 (3.6)
Retinopathy	1124 (8.2)	873 (7.6)	743 (5.8)	2288 (6.3)
Neuropathy	1937 (14.1)	1681 (14.6)	1175 (9.1)	3848 (10.6)
Bacterial foot infection	172 (1.3)	163 (1.4)	161 (1.3)	399 (1.1)
Fungal foot infection	15 (0.1)	12 (0.1)	21 (0.2)	41 (0.1)
Osteomyelitis	44 (0.3)	47 (0.4)	38 (0.3)	103 (0.3)
Lower limb amputation	96 (0.7)	85 (0.7)	69 (0.5)	238 (0.7)
Diabetic foot ulcer	644 (4.7)	606 (5.3)	397 (3.1)	1327 (3.7)
Signs of hypovolemia in the year before index date				
Volume depletion	176 (1.3)	163 (1.4)	277 (2.2)	935 (2.6)
Anuria or oliguria	5 (0.0)	< 5 (0.0)	7 (0.1)	11 (0.0)
Hypotension	110 (0.8)	66 (0.6)	100 (0.8)	353 (1.0)
Drugs prescriptions in the 6 months before index date				
Insulin	2513 (18.3)	1977 (17.2)	107 (0.8)	1186 (3.3)
Loop diuretics	1464 (10.7)	1225 (10.6)	1250 (9.7)	4659 (12.9)
Thiazide diuretics	1880 (13.7)	1237 (10.7)	1397 (10.9)	4333 (12.0)
Potassium sparing diuretics	824 (6.0)	580 (5.0)	548 (4.3)	1863 (5.2)
Beta-blockers	3532 (25.7)	2121 (18.4)	2544 (19.8)	8372 (23.2)
Calcium-channel blockers	3849 (28.0)	2294 (19.9)	2350 (18.3)	8557 (23.7)
ACE inhibitors	4609 (33.6)	2757 (23.9)	3037 (23.6)	10,327 (28.6)
ARBs	4502 (32.8)	2595 (22.5)	2503 (19.5)	9006 (24.9)
Digoxin	367 (2.7)	201 (1.7)	388 (3.0)	1297 (3.6)
Organic nitrates	493 (3.6)	280 (2.4)	346 (2.7)	1170 (3.2)
Antithrombotic agents	5198 (37.8)	2939 (25.5)	3423 (26.6)	11,914 (33.0)
Lipid lowering drugs	9808 (71.4)	5559 (48.3)	6085 (47.4)	21,450 (59.3)

*SGLT2-I* sodium-glucose co-transporter-2 inhibitor, *GLP1-RA* glucagon-like peptide-1 receptor agonist, *SU* sulfonylurea, *DPP4-I* dipeptidyl peptidase-4 inhibitor, *IQR* inter quartile range, *SD* standard deviation, *ACE* angiotensin converting enzyme, *ARB* angiotensin II receptor blocker

^a^Data are presented as number (%) of individuals, unless stated otherwise

^b^Combined users (N = 230) are not shown

^c^Basic = basic school/high school education: 7–12 years of primary, secondary, and grammar school education; Secondary = vocational education, 10–12 years of education; Higher = a university degree or an examination in another higher institution requiring an average of 13 years or more

In total we observed 564 LLAs (367 of which were forefoot amputations, i.e. through or below the metatarsal bones) during follow-up. The current SGLT2-I exposure time comprised of 49.8% empagliflozin, 47.3% dapagliflozin, 2.9% canagliflozin, and < 0.1% ertugliflozin. The current GLP1-RA exposure time comprised of 93.2% liraglutide, 3.3% dulaglutide, 1.7% exenatide, 1.6% semaglutide, and 0.3% lixisenatide. Table 2 shows that the risk of LLA was similar with current SGLT2-I use compared to current SU use (adjusted HR 1.10; 95% confidence interval [CI] 0.71–1.70). This finding remained consistent after stratification by sex and age groups. Similarly, the risk of forefoot amputations was similar with SGLT2-I use compared to SU use (adjusted HR 0.90 95%CI 0.52–1.57). From Table 3 it becomes apparent that the risk of LLA remains similar after stratification by concomitant antihypertensive use. Wald tests confirmed there were no differences between the groups of these stratifications. There were no LLAs with both SGLT2-I use exposure and a record of signs of hypovolaemia (volume depletion, anuria/oliguria, hypotension), so we were unable to study the potential effects of these conditions (data not shown). The risk of LLA was markedly elevated with the presence of PAD. Table 4 indicates that a higher cumulative dose but the duration of use of an SGLT2-I is associated with a higher risk of LLA.

**Table 2:** Risk of LLA in current SGLT2-I use compared to current SU use, stratified by sex and age

	Number of LLAs (N = 564)	IR (/1000 PY	Age/sex adjusted HR (95%CI)	Fully adjusted HR (95%CI)
Current SU use	54	2.14	Reference	
Current DPP4-I use	149	2.18	1.01 (0.74–1.38)	0.89 (0.65–1.22)^d^
Current combined use ^a^	90	2.48	1.28 (0.91–1.80)	0.99 (0.70–1.41)^d^
Current other NIGLD use	59	1.87	0.92 (0.64–1.34)	0.76 (0.53–1.11)^d^
Current SGLT2-I use	36	2.30	1.24 (0.81–1.91)	1.10 (0.71–1.70)^d^
By sex^b^				
Males	32	3.30	1.22 (0.77–1.94)	1.12 (0.70–1.79)^e^
Females	< 5	0.67	1.21 (0.36–4.02)	0.94 (0.28–3.15)^f^
By age (years)^c^				
18–49	< 5	1.07	2.77 (0.28–27.08)	2.26 (0.23–22.09)^g^
50–59	11	2.31	1.49 (0.59–3.77)	1.02 (0.40–2.61)^h^
60–69	13	2.65	1.48 (0.68–3.22)	1.25 (0.57–2.75)^i^
70 +	9	2.84	0.94 (0.44–2.00)	1.10 (0.51–2.34)^d^

The models have also been adjusted for current GLP1-RA use (55 LLAs) and past NIGLD use (121 LLAs) (not shown). In the stratifications, the number of included confounders in each Cox regression model was based on a maximum of one confounder per ten events [24]

*LLA* lower limb amputation, *IR* incidence rate, *PY* person years, *HR* hazard ratio, *CI* confidence interval, *NIGLD* non-insulin glucose lowering drug, *SU* sulfonylurea, *DPP4-I* dipeptidyl peptidase-4 inhibitor, *SGLT2-I* sodium-glucose co-transporter-2 inhibitor

^a^Combined use of at least two of the following NIGLDs: SGLT2-I and/or GLP1-RA and/or SU and/or DPP4-I

^b^Compared with controls of the same sex

^c^Compared with controls in the same age category

^d^Adjusted for age; sex; diabetes duration; income category; history of diabetic foot ulcer, neuropathy, atherosclerosis, renal disease, osteomyelitis, retinopathy, hypertension, heart failure, ischaemic heart disease, or peripheral arterial disease; and the use of insulin, beta-blockers, angiotensin receptor blockers, antithrombotic agents, or lipid lowering drugs in the 6 months before the start of the exposure interval

^e^Adjusted for age; diabetes duration; income category; history of diabetic foot ulcer, neuropathy, atherosclerosis, renal disease, osteomyelitis, retinopathy, hypertension, heart failure, ischaemic heart disease, or peripheral arterial disease; and the use of potassium sparing diuretics beta-blockers, angiotensin receptor blockers, antithrombotic agents, or lipid lowering drugs in the 6 months before the start of the exposure interval

^f^Adjusted for age; diabetes duration; history of diabetic foot ulcer, neuropathy, atherosclerosis, or renal disease; and the use of antithrombotic agents in the 6 months before the start of the exposure interval

^g^Adjusted for age; sex; and diabetes duration

^h^Adjusted for age; sex; diabetes duration; history of diabetic foot ulcer, neuropathy, atherosclerosis, or renal disease; and the use of antithrombotic agents in the 6 months before the start of the exposure interval

^i^Adjusted for age; sex; diabetes duration; income category; history of diabetic foot ulcer, neuropathy, atherosclerosis, renal disease, osteomyelitis, retinopathy, hypertension, or heart failure; and the use of antithrombotic agents, or lipid lowering drugs in the 6 months before the start of the exposure interval

**Table 3:** Risk of LLA in current SGLT2-I use compared to current SU use, stratified by concomitant antihypertensive use, and history of peripheral arterial disease

	Number of LLAs (N = 564)	IR (/1000 PY	Age/sex adjusted HR (95%CI)	Fully adjusted HR (95%CI)	p-value Wald test
Current SU use	54	2.14	Reference		
Current SGLT2-I use	36	2.30	1.24 (0.81–1.91)	1.10 (0.71–1.70)^a^	
By concomitant antihypertensive use in the previous 3 months					
Diuretics					
Yes	13	2.41	1.22 (0.66–2.25)	0.98 (0.53–1.81)^b^	0.60
No	23	2.24	1.25 (0.76–2.06)	1.17 (0.71–1.94)^b^	
Drugs acting on RAAS					
Yes	24	2.84	1.44 (0.88–2.35)	1.23 (0.75–2.02)^c^	0.35
No	12	1.67	0.97 (0.52–1.83)	0.88 (0.47–1.67)^c^	
By history of PAD					
Yes	11	17.57	7.21 (3.75–13.86)	2.24 (1.15–4.36)^d^	0.01
No	25	1.67	0.91 (0.56–1.47)	0.92 (0.56–1.49)^d^	

The models have also been adjusted for current DPP4-I use (149 LLAs), current combined use (90 LLAs), current other NIGLD use (59 LLAs), current GLP1-RA use (55 LLAs), and past NIGLD use (121 LLAs) (not shown)

*LLA* lower limb amputation, *IR* incidence rate, *PY* person years, *HR* hazard ratio, *CI* confidence interval, *NIGLD* non-insulin glucose lowering drug, *SU* sulfonylurea, *DPP4-I* dipeptidyl peptidase-4 inhibitor, *SGLT2-I* sodium-glucose co-transporter-2 inhibitor, *RAAS* renin–angiotensin–aldosterone system, *PAD* peripheral arterial disease

^a^Adjusted for age; sex; diabetes duration; income category; history of diabetic foot ulcer, neuropathy atherosclerosis, renal disease, osteomyelitis, retinopathy, hypertension, heart failure, ischaemic heart disease, or peripheral arterial disease; and the use of antithrombotic agents, lipid lowering drugs, potassium sparing diuretics, beta blockers, or angiotensin receptor blockers in the 6 months before the start of the exposure interval

^b^Adjusted for age; sex; diabetes duration; income category; history of diabetic foot ulcer, neuropathy atherosclerosis, renal disease, osteomyelitis, retinopathy, hypertension, heart failure, ischaemic heart disease, or peripheral arterial disease; and the use of antithrombotic agents, lipid lowering drugs, beta blockers, or angiotensin receptor blockers in the 6 months before the start of the exposure interval

^c^Adjusted for age; sex; diabetes duration; income category; history of diabetic foot ulcer, neuropathy atherosclerosis, renal disease, osteomyelitis, retinopathy, hypertension, heart failure, ischaemic heart disease, or peripheral arterial disease; and the use of antithrombotic agents, lipid lowering drugs, potassium sparing diuretics, or beta blockers in the 6 months before the start of the exposure interval

^d^Adjusted for age; sex; diabetes duration; income category; history of diabetic foot ulcer, neuropathy atherosclerosis, renal disease, osteomyelitis, retinopathy, hypertension, heart failure, or ischaemic heart disease; and the use of antithrombotic agents, lipid lowering drugs, potassium sparing diuretics, beta blockers, or angiotensin receptor blockers in the 6 months before the start of the exposure interval

**Table 4:** Risk of LLA in current SGLT2-I use compared to current SU use, stratified by cumulative dose and continuous duration of use

	Number of LLAs (N = 564)	IR (/1000 PY	Age/sex adjusted HR (95%CI)	Fully adjusted HR (95%CI)^a^
Current SU use	54	2.14	Reference	
Current SGLT2-I use	36	2.30	1.24 (0.81–1.91)	1.10 (0.71–1.70)
By cumulative dose				
< 250 DDDs	15	2.28	1.26 (0.71–2.24)	1.15 (0.64–2.05)
250–1200 DDDs	10	1.46	0.79 (0.40–1.55)	0.68 (0.34–1.34)
≥ 1200 DDDs	11	4.92	2.58 (1.34–5.00)	2.24 (1.16–4.36)
By continuous duration of use				
1–160 days	15	2.42	1.32 (0.74–2.36)	1.20 (0.67–2.15)
161–365 days	7	1.68	0.91 (0.41–2.01)	0.83 (0.37–1.83)
> 365 days	14	3.18	1.68 (0.92–3.04)	1.38 (0.76–2.52)

The models have also been adjusted for current DPP4-I use (149 LLAs), current combined use (90 LLAs), current other NIGLD use (59 LLAs), current GLP1-RA use (55 LLAs), and past NIGLD use (121 LLAs) (not shown)

*LLA* lower limb amputation, *IR* incidence rate, *PY* person years, *HR* hazard ratio, *CI* confidence interval, *NIGLD* non-insulin glucose lowering drug, *SU* sulfonylurea, *DPP4-I* dipeptidyl peptidase-4 inhibitor, *SGLT2-I* sodium-glucose co-transporter-2 inhibitor, *DDD* daily defined dose

^a^Adjusted for age; sex; diabetes duration; income category; history of diabetic foot ulcer, neuropathy, atherosclerosis, osteomyelitis, retinopathy, hypertension, heart failure, ischaemic heart disease, or peripheral arterial disease; and the use of antithrombotic agents, lipid lowering drugs, potassium sparing diuretics, beta blockers, or angiotensin receptor blockers in the 6 months before the start of the exposure interval

The risk of LLAs was lower (adjusted HR 0.57; 95%CI 0.39–0.84) with current GLP1-RA use compared to current SU use (Table 5). After stratification, this finding remained apparent in females (9 LLAs) but not in males (46 LLAs) nor in any of the age categories (number of LLAs ranging from 6 to 18 per category). The lower risk was also observed when limiting the outcome to forefoot amputations only (adjusted HR 0.57; 95%CI 0.36–0.92). Table 6 shows that the lower risk of LLA remained consistent after stratifying by concomitant antihypertensive use. There was a limited number of LLAs with both GLP1-RA use and a record of signs of hypovolaemia (volume depletion, anuria/oliguria, hypotension), so we were unable to study the potential effects of these conditions (data not shown). Stratification by history of PAD showed a large difference in IR (yes 9.86, no 1.10 LLAs /1000 PY). Table 7 shows the association between longer duration of GLP1-RA use and the risk of LLA, which was mostly apparent in the highest continuous duration of use category (> 365 days, adjusted HR 0.44; 95%CI 0.27–0.72).

**Table 5:** Risk of LLA in current GLP1-RA use compared to current SU use, stratified by sex and age

	Number of LLAs (N = 564)	IR (/1000 PY	Age/sex adjusted HR (95%CI)	Fully adjusted HR (95%CI)
Current SU use	54	2.14	Reference	
Current DPP4-I use	149	2.18	1.01 (0.74–1.38)	0.89 (0.65–1.22)^d^
Current combined use^a^	90	2.48	1.28 (0.91–1.80)	1.00 (0.71–1.42)^d^
Current other NIGLD use	59	1.87	0.92 (0.64–1.34)	0.80 (0.55–1.17)^d^
Current GLP1-RA use	55	1.51	0.88 (0.60–1.29)	0.57 (0.39–0.84)^d^
By sex^b^				
Males	46	2.28	0.88 (0.58–1.33)	0.57 (0.38–0.88)^e^
Females	9	0.56	0.94 (0.37–2.39)	0.53 (0.21–1.38)^f^
By age (years)^c^				
18–49	6	0.84	2.82 (0.34–23.42)	2.09 (0.25–17.46)^g^
50–59	13	1.20	0.87 (0.36–2.10)	0.49 (0.20–1.19)^h^
60–69	18	1.55	0.94 (0.46–1.93)	0.50 (0.24–1.04)^i^
70 +	18	2.70	0.90 (0.50–1.61)	0.64 (0.35–1.17)^j^

The models have also been adjusted for current SGLT2-I use (36 LLAs) and past NIGLD use (121 LLAs) (not shown). In the stratifications, the number of included confounders in each Cox regression model was based on a maximum of one confounder per ten events [24]

*LLA* lower limb amputation, *IR* incidence rate, *PY* person years, *HR* hazard ratio, *CI* confidence interval, *NIGLD* non-insulin glucose lowering drug, *SU* sulfonylurea, *DPP4-I* dipeptidyl peptidase-4 inhibitor, *GLP1-RA* glucagon-like peptide-1 receptor agonist

^a^Combined use of at least two of the following NIGLDs: SGLT2-I and/or GLP1-RA and/or SU and/or DPP4-I

^b^Compared with controls of the same sex

^c^Compared with controls in the same age category

^d^Adjusted for age; sex; diabetes duration; income category; immigrant status; education; history of diabetic foot ulcer, neuropathy, atherosclerosis, peripheral arterial disease, hypertension, retinopathy heart failure, hyperlipidaemia, ischaemic heart disease, osteomyelitis, renal disease, pulmonary heart disease, or bacterial foot infection; and the use of loop diuretics, antithrombotic agents, potassium sparing diuretics, beta blockers, lipid lowering drugs, angiotensin receptor blockers, digoxin, angiotensin converting enzyme inhibitors, or calcium channel blockers in the 6 months before the start of the exposure interval

^e^Adjusted for age; diabetes duration; income category; immigrant status; education; history of diabetic foot ulcer, neuropathy, atherosclerosis, peripheral arterial disease, hypertension, retinopathy heart failure, hyperlipidaemia, ischaemic heart disease, osteomyelitis, renal disease, pulmonary heart disease, or bacterial foot infection; and the use of loop diuretics, antithrombotic agents, potassium sparing diuretics, beta blockers, lipid lowering drugs, angiotensin receptor blockers, digoxin, angiotensin converting enzyme inhibitors, or calcium channel blockers in the 6 months before the start of the exposure interval

^f^Adjusted for age; diabetes duration; history of diabetic foot ulcer, neuropathy, atherosclerosis, or peripheral arterial disease; and the use of loop diuretics in the 6 months before the start of the exposure interval

^g^Adjusted for age; sex; and diabetes duration

^h^Adjusted for age; sex; diabetes duration; history of diabetic foot ulcer, neuropathy, atherosclerosis, or peripheral arterial disease; and the use of loop diuretics in the 6 months before the start of the exposure interval

^i^Adjusted for age; sex; diabetes duration; income category; history of diabetic foot ulcer, neuropathy, atherosclerosis, peripheral arterial disease, hypertension, retinopathy, or heart failure; and the use of loop diuretics, antithrombotic agents, or potassium sparing diuretics in the 6 months before the start of the exposure interval

^j^Adjusted for age; sex; diabetes duration; income category; history of diabetic foot ulcer, neuropathy, atherosclerosis, peripheral arterial disease, hypertension, retinopathy, heart failure, hyperlipidaemia, or ischaemic heart disease; and the use of loop diuretics, antithrombotic agents, potassium sparing diuretics, beta blockers, lipid lowering drugs, or angiotensin receptor blockers in the 6 months before the start of the exposure interval

**Table 6:** Risk of LLA in current GLP1-RA use compared to current SU use, stratified by concomitant antihypertensive use, and history of peripheral arterial disease

	Number of LLAs (N = 564)	IR (/1000 PY	Age/sex adjusted HR (95%CI)	Fully adjusted HR (95%CI)	p-value Wald test
Current SU use	54	2.14	Reference		
Current GLP1-RA use	55	1.51	0.88 (0.60–1.29)	0.57 (0.39–0.84)^a^	
By concomitant antihypertensive use in the previous 3 months					
Diuretics					
Yes	27	1.81	0.96 (0.60–1.52)	0.55 (0.34–0.88)^b^	0.74
No	28	1.31	0.82 (0.52–1.30)	0.60 (0.38–0.96)^b^	
Drugs acting on RAAS					
Yes	34	1.64	0.88 (0.57–1.35)	0.57 (0.36–0.88)^c^	0.98
No	21	1.35	0.89 (0.53–1.47)	0.57 (0.34–0.95)^c^	
By history of PAD					
Yes	17	9.86	4.05 (2.35–7.00)	0.94 (0.54–1.65)^d^	0.04
No	38	1.10	0.65 (0.43–0.99)	0.51 (0.33–0.77)^d^	

The models have also been adjusted for current DPP4-I use (149 LLAs), current combined use (90 LLAs), current other NIGLD use (59 LLAs), current SGLT2-I use (36 LLAs) and past NIGLD use (121 LLAs) (not shown)

*LLA* lower limb amputation, *IR* incidence rate, *PY* person years, *HR* hazard ratio, *CI* confidence interval, *NIGLD* non-insulin glucose lowering drug, *SU* sulfonylurea, *DPP4-I* dipeptidyl peptidase-4 inhibitor, *GLP1-RA* glucagon-like peptide-1 receptor agonist

^a^Adjusted for age; sex; diabetes duration; income category; immigrant status; education; history of diabetic foot ulcer, neuropathy, atherosclerosis, peripheral arterial disease, hypertension, retinopathy, heart failure, hyperlipidaemia, ischaemic heart disease, osteomyelitis, renal disease, pulmonary heart disease, or bacterial foot infection; and the use of loop diuretics, antithrombotic agents, potassium sparing diuretics, beta blockers, lipid lowering drugs, angiotensin receptor blockers, digoxin, angiotensin converting enzyme blockers, or calcium channel blockers in the 6 months before the start of the exposure interval

^b^Adjusted for age; sex; diabetes duration; income category; immigrant status; education; history of diabetic foot ulcer, neuropathy, atherosclerosis, peripheral arterial disease, hypertension, retinopathy, heart failure, hyperlipidaemia, ischaemic heart disease, osteomyelitis, renal disease, pulmonary heart disease, or bacterial foot infection; and the use of antithrombotic agents, beta blockers, lipid lowering drugs, angiotensin receptor blockers, digoxin, angiotensin converting enzyme blockers, or calcium channel blockers in the 6 months before the start of the exposure interval

^c^Adjusted for age; sex; diabetes duration; income category; immigrant status; education; history of diabetic foot ulcer, neuropathy, atherosclerosis, peripheral arterial disease, hypertension, retinopathy, heart failure, hyperlipidaemia, ischaemic heart disease, osteomyelitis, renal disease, pulmonary heart disease, or bacterial foot infection; and the use of loop diuretics, antithrombotic agents, potassium sparing diuretics, beta blockers, lipid lowering drugs, digoxin, or calcium channel blockers in the 6 months before the start of the exposure interval

^d^Adjusted for age; sex; diabetes duration; income category; immigrant status; education; history of diabetic foot ulcer, neuropathy, atherosclerosis, hypertension, retinopathy, heart failure, hyperlipidaemia, ischaemic heart disease, osteomyelitis, renal disease, pulmonary heart disease, or bacterial foot infection; and the use of loop diuretics, antithrombotic agents, potassium sparing diuretics, beta blockers, lipid lowering drugs, angiotensin receptor blockers, digoxin, angiotensin converting enzyme blockers, or calcium channel blockers in the 6 months before the start of the exposure interval

**Table 7:** Risk of LLA in current GLP1-RA use compared to current SU use, stratified by cumulative dose and continuous duration of use

	Number of LLAs (N = 564)	IR (/1000 PY	Age/sex adjusted HR (95%CI)	Fully adjusted HR (95%CI)^a^
Current SU use	54	2.14	Reference	
Current GLP1-RA use	55	1.51	0.88 (0.60–1.29)	0.57 (0.39–0.84)
By cumulative dose				
< 250 DDDs	15	1.48	0.92 (0.52–1.64)	0.65 (0.37–1.17)
250–1200 DDDs	20	1.09	0.64 (0.38–1.07)	0.42 (0.25–0.71)
≥ 1200 DDDs	20	2.54	1.36(0.81–2.29)	0.76 (0.45–1.30)
By continuous duration of use				
1–160 days	21	2.26	1.42 (0.85–2.35)	0.95 (0.57–1.59)
161–365 days	11	1.37	0.81 (0.42–1.55)	0.53 (0.28–1.03)
> 365 days	23	1.27	0.71 (0.43–1.15)	0.44 (0.27–0.72)

The models have also been adjusted for current DPP4-I use (149 LLAs), current combined use (90 LLAs), current other NIGLD use (59 LLAs), current SGLT2-I use (36 LLAs) and past NIGLD use (121 LLAs) (not shown)

*LLA* lower limb amputation, IR incidence rate, *PY* person years, *HR* hazard ratio, *CI* confidence interval, *NIGLD* non-insulin glucose lowering drug, *SU* sulfonylurea, *DPP4-I* dipeptidyl peptidase-4 inhibitor, *GLP1-RA* glucagon-like peptide-1 receptor agonist, *DDD* daily defined dose

^a^Adjusted for age; sex; diabetes duration; income category; immigrant status; education; history of diabetic foot ulcer, neuropathy, atherosclerosis, peripheral arterial disease, hypertension retinopathy, heart failure, hyperlipidaemia, ischaemic heart disease, osteomyelitis, renal disease, pulmonary heart disease, or bacterial foot infection; and the use of loop diuretics, antithrombotic agents, potassium sparing diuretics, lipid lowering drugs, angiotensin receptor blockers, digoxin, angiotensin converting enzyme inhibitors, or calcium channel blockers in the 6 months before the start of the exposure interval

We observed 1,524 DFUs during follow-up. Neither SGLT2-I use nor GLP1-RA use were associated with the risk of DFU (adjusted HR 1.18; 95%CI 0.90–1.55 and adjusted HR 1.08; 95%CI 0.87–1.35, respectively).

In the first sensitivity analysis in which we changed the reference group from SU to DPP4-I, we obtained similar results to our main analyses. There was no significant association of SGLT2-I use with the risk of LLA (adjusted HR 1.23; 95%CI 0.85–1.79), forefoot amputation (adjusted HR 0.98; 95%CI 0.61–1.58) and DFU (adjusted HR 1.06; 95%CI 0.85–1.33) compared to SU use. GLP1 use was associated with a lower risk of LLA (adjusted HR 0.64; 95%CI 0.47–0.88) and forefoot amputation (adjusted HR 0.63; 95%CI 0.43–0.92), and a similar risk of DFU (adjusted HR 1.00; 95%CI 0.84–1.17) compared to SU use (Additional file 1: Table S1). In the second sensitivity analysis in which we excluded individuals with a history of LLA at baseline, we observed 470 LLAs during follow up and the findings remained consistent. There were 29 LLAs with current SGLT2-I use (adjusted HR 1.05; 95%CI 0.65–1.69) and 43 with current GLP1-RA use (adjusted HR 0.51; 95%CI 0.33–0.78) (Additional file 1: Table S2). Finally, the addition of number of concomitant glucose-lowering drugs and number of bed days as confounders, did not alter the results. The resulting HRs were 0.97 (95%CI 0.62–1.52) with current SGLT2-I use and 0.51 (0.34–0.76) with current GLP1-RA use.

## Discussion

The results of this nation-wide cohort study indicated that SGLT2-I use was not associated with a higher risk of LLA compared to SU. This finding remained consistent after stratification by age, sex, concomitant antihypertensive use, history of PAD, and continuous duration of use categories. GLP1-RA use, however, was associated with a lower risk of LLA. This risk reduction was particularly apparent in men, but also remained consistent after stratification by concomitant antihypertensive use and history of PAD. Stratification by continuous duration of use indicated that increasing duration of GLP1-RA use was associated with a lower risk of LLA. Both SGLT2-Is and GLP1-RAs did not alter the risk of DFU.

The findings of previous studies might have been influenced by choosing GLP1-RAs as a reference group when evaluating the risk of LLA. In the current study, we took another approach at evaluating the association between SGLT2-Is and GLP1-RAs and LLA, by separately comparing them to an active comparator. The results of this study are in line with meta-analyses in which no higher risk of LLA was observed with SGLT2-I use compared to other glucose-lowering drugs and placebo [9, 11, 28, 29]. One meta-analysis of RCTs observed a slightly higher risk of LLA with SGLT2-I versus placebo [30], but this risk was driven by canagliflozin, the SGLT2-I which was hardly used by the participants in the current study. Another meta-analysis reported no evidence was found for an effect of SGLT2-I or GLP1-RA on LLA, but there was high uncertainty regarding these results based on Grading of Recommendations, Assessment, Development and Evaluations (GRADE) [31]. Our results are also in line with a recent meta-analysis of observational studies, with a hypothesis about SGLT2-I versus GLP1-RA effects that was similar to ours [32]. Indeed, the author reported a lower risk of LLA with GLP1-RA compared to SGLT2-I. Unfortunately none of the studies included were designed to investigate LLAs and both direct and indirect comparisons were made. Moreover, in contrast to our study, the comparison of SGLT2-I with GLP1-RA did not allow for distinction between the effects exerted by the drug classes separately. Finally, our results are in line with several cohort studies which reported no higher risk of LLA with SGLT2-Is [17, 33, 34] and a lower risk of LLA with GLP1-RAs [30, 35].

Overall, the available evidence for the association between SGLT2-I use and GLP1-RA use and the risk of LLA is limited, has high uncertainty [31], and the studies are highly heterogeneous [36]. It is important to note that the choice of reference group varies among the abovementioned studies. These differences could play a large role in the conflicting nature of the reported results. Some studies investigating the risk of LLA with SGLT2-I have chosen GLP1-RAs as the reference group. Several of these studies report no difference [33, 34, 37], whereas others report a higher risk with SGLT2-I [12, 13, 38, 39]. In order to compare the current study with these previous findings, we performed a post-hoc analysis comparing SGLT2-I directly to GLP1-RAs. We observed an increased risk with current SGLT2-I use versus current GLP1-RA use (adjusted HR 1.91; 95%CI 1.25–2.92), which is in line with our expectations, as well as the previous studies. The comparison of SGLT2-I with SU has only been reported in two cohort studies; one reporting a similar risk of LLA [17] and one reporting a lower risk with SGLT2-Is [40]. The discordant findings between the current study and the study of Dawwas et al. [40], may be explained by a difference in populations included in the used data sources. The population included in the study by Dawwas et al. consisted of privately insured individuals with employer-sponsored health insurance programs and retirees whereas our study includes the complete Danish population. Furthermore, a shorter average follow-up time (median of 131 days) was reported potentially explaining the lower IRs found in the study by Dawwas et al. As a result of these differences a part of the population at risk of LLA is possibly not captured and potential harmful or protective effects on LLA may not have been visible yet. The comparison of GLP1-RA with SU has not been reported before, most likely because in most countries these drugs are not used in a similar disease stage. In Denmark, however, both these drug classes, as well as SGLT2-Is, are recommended as second-line treatment [24, 41].

The effect size of current GLP1-RA on the risk of LLA was remarkably large in this study, with approximately 40% risk reduction in our analyses. In order to rule out potential issues causing such an unexpectedly large effect, we further explored this finding. We found no disproportionality regarding person time, IRs and unadjusted HRs. Since the large effect size was particularly apparent in the fully adjusted HRs, we evaluated various aspects of the confounders included in the models. We ruled out multicollinearity and that individual confounders had an unexpected effect on the beta coefficient in the model, paying special attention to insulin use. The results remained consistent in models with and without correction for concomitant insulin use. Furthermore, we explored the possibility of distortion by unmeasured confounding. We calculated the e-value, which estimates the strength of unmeasured confounding required to nullify the observed effect [42, 43]. Using the observed HR of 0.57(95%CI 0.39–0.84) (Table 5), the e-value for the HR point estimate was 2.9 and the e-value for the upper bound of the CI was 1.7. This means that the unmeasured confounding would have to threefold the risk in order to nullify the HR, or have a strength of 1.7 to shift the CI to overlap the null. Although some highly intensive treatment interventions have shown to reduce the risk of LLA up to threefold compared to usual care [44], we don’t expect these types of strategies would be implemented differentially between the evaluated drug classes in this study. So, although we are unsure of the actual size of the effect, substantial unmeasured confounding is required to nullify the observed effect. Therefore, it is safe to say our data suggest GLP1-RA use has a protective effect against LLA.

Many mechanisms through which drugs could alter the risk of LLA have been proposed. Although the effect of SGLT2-Is on LLA is highly debated, the most commonly proposed mechanism for this potential effect is hypovolaemia. It has been proposed that the increased sodium excretion and osmotic diuresis caused by increased glucose excretion due to SGLT2-I use, could lead to reduced peripheral tissue perfusion, necrosis and finally requiring amputation [16]. This hypovolaemia-based mechanism is supported by the finding of an higher risk of LLA in T2D patients using diuretics compared to those not using diuretics [45] and the association of a reduction in body weight and blood pressure with lower limb complications in patients using SGLT2-Is [46]. However, in the current study we did not observe a higher risk of LLA with SGLT2-Is, nor did we find LLAs with current SGLT2-I exposure and the presence of signs of hypovolaemia. Moreover, we did not observe a change in the risk of LLA with or without concomitant diuretic or RAAS inhibitor use.

The evidence regarding the role of GLP1 in the processes leading to LLA is limited. We speculate the pathways involved could be either via effects on wound healing or PAD. Findings from basic research have suggested that GLP1-RAs may promote DFU healing by boosting various physiological processes involved in diabetic wound healing [47, 48]. This would prevent a non-healing DFU to require amputation as treatment. The other possible pathway is via PAD, since GLP1 exhibits positive vascular effects [49, 50]. In the current study, we did not observe a lower risk of LLA in patients with a history of PAD; the risk was elevated in this high risk group with both SGLT2-I and GLP1-RA use. Based on this finding, it is more likely that wound healing might play a role in the protective effect of GLP1-RAs. We observed no change in risk of DFU with GLP1-RA use, but we were unable to study the healing of DFUs, the phase in which GLP1-RAs might potentially exert their effect. Therefore, further study is required to look into the mechanism underlying the protective effect of GLP1-RAs on LLA.

This study has several strengths. The availability of nation-wide real-world data and the completeness of the Danish Health Registries allowed for more accurate recording of LLAs. Moreover, the database allowed us to correct for a comprehensive set of confounders, such as socio-economic variables, which play an important role in the development of diabetic foot issues [51]. Another strength is the consistency of the results across various stratifications, showing that the models are robust and the findings reliable. Additionally, the findings remained consistent in the sensitivity analyses in which we changed the reference group to another second-line treatment option, and another in which we excluded individuals with LLA before index date. This further supports the fact that our models are robust and produced reliable findings. Moreover, we critically assessed potential causes for overestimation of the effect size observed with current GLP1-RA exposure but found no indications of augmentation of the effect size. Finally, a post-hoc analysis comparing current SGLT2-I use directly to current GLP1-RA use, confirmed that the use of GLP1-RA use as a reference group indeed leads to the observation of an increased risk of LLA.

Despite the availability of nation-wide data, the observational study design requires us to consider several limitations when interpreting the results. The results might have been influenced by unmeasured confounding by factors which were not available in the dataset, such as glycosylated haemoglobin A1c (HbA1c) values, body mass index, and smoking status. However, the e-value we calculated indicated a considerable amount of unmeasured confounding would be required to nullify the observed effect. Another limitation to take into account is the limited number of events. Due to the small number of events, we were unable to study various signs of hypovolaemia, and to evaluate substance-specific effects instead of drug-class effects. The power issue caused by the limited number of events is also reflected in some stratifications. The point estimate of the protective effect of GLP1-RAs is similar in men and women, but the 95% CI indicates significance only in men. This is also the case in the highest age stratum (70 +) of current GLP1-RA use. Although the point estimate for the risk of LLA with SGLT2-I versus SU was close to 1, the width of the CI also allows for a true risk to be either higher or lower.

Moreover, records of DFU might be less accurate then records of LLA, since amputation procedures are recorded in the hospital database, whereas a DFU record relies on a registration of a diagnosis. Therefore the data might suffer from underrecording of DFU diagnoses.

In conclusion, this nation-wide cohort study indicates that SGLT2-I use is not associated with a higher risk of LLA compared to SU use. However, GLP1-RA use was associated with a lower risk of LLA. The risk of LLA was higher in patients with PAD, irrespective of SGLT2-I or GLP1-RA use. The results of this study add to the growing body of evidence that SGLT2-I use is not associated with a higher risk of LLA, and help to explain the observed harmful effect when comparing this drug class to GLP1-RAs. Previous studies reporting a higher risk of LLA with SGLT2-I use compared to GLP1-RA use might have been looking at a protective GLP1-RA-effect, instead of a harmful SGLT2-I-effect. The current findings put forward GLP1-RAs as a potential treatment strategy to reduce the risk of LLA in patients with T2D, but further study is required to confirm this potential effect and the underlying mechanisms.

## Supplementary Information

**Additional file 1: Figure S1** Graphical depiction [23] of the analysis of the study cohort. **Table S1** Risk of LLA, forefoot amputation and DFU in current use of different NIGLDs compared to DPP4-I use (sensitivity analysis 1). **Table S2** Risk of LLA in current use of different NIGLDs compared to SU use excluding LLA history at baseline (sensitivity analysis 2).

## Data Availability

The data that support the findings of this study were made available from Statistics Denmark for this specific study in an authorised research and analysis environment, and so are not publicly available.

## Abbreviations

ACE: Angiotensin converting enzyme
ARB: Angiotensin II receptor blocker
ATC: Anatomical therapeutic chemical
CI: Confidence interval
DDD: Daily defined doses
DFU: Diabetic foot ulcer
DPP4-I: Dipeptidyl peptidase-4 inhibitor
GLP1-RA: Glucagon-like peptide-1 receptor agonist
HR: Hazard ratio
ICD: International classification of diseases
IQR: Inter quartile range
IR: Incidence rate
LLA: Lower limb amputation
NIGLD: Non-insulin glucose lowering drug
PAD: Peripheral arterial disease
PY: Person years
RAAS: Renin–angiotensin–aldosterone system
SGLT2-I: Sodium-glucose co-transporter-2 inhibitor
SU: Sulfonylurea
T2D: Type 2 diabetes
