# Supplementary material for: The use of sodium-glucose co-transporter-2 inhibitors or glucagon-like peptide-1 receptor agonists versus sulfonylureas and the risk of lower limb amputations: a nation-wide cohort study
Source: Cardiovasc Diabetol. 2023 Jun 29;22:160. doi: 10.1186/s12933-023-01897-2 (PMC10311702; doi:10.1186/s12933-023-01897-2)
Supplement: Supplementary file 1 — Additional file 1: Figure S1 Graphical depiction [23] of the analysis of the study cohort. Table S1 Risk of LLA, forefoot amputation and DFU in current use of different NIGLDs compared to DPP4-I use (sensitivity analysis 1). Table S2 Risk of LLA in current use of different NIGLDs compared to SU use excluding LLA history at baseline (sensitivity analysis 2). [file 12933_2023_1897_MOESM1_ESM.docx]

**Figure S1** Graphical depiction (23) of the analysis of the study cohort.


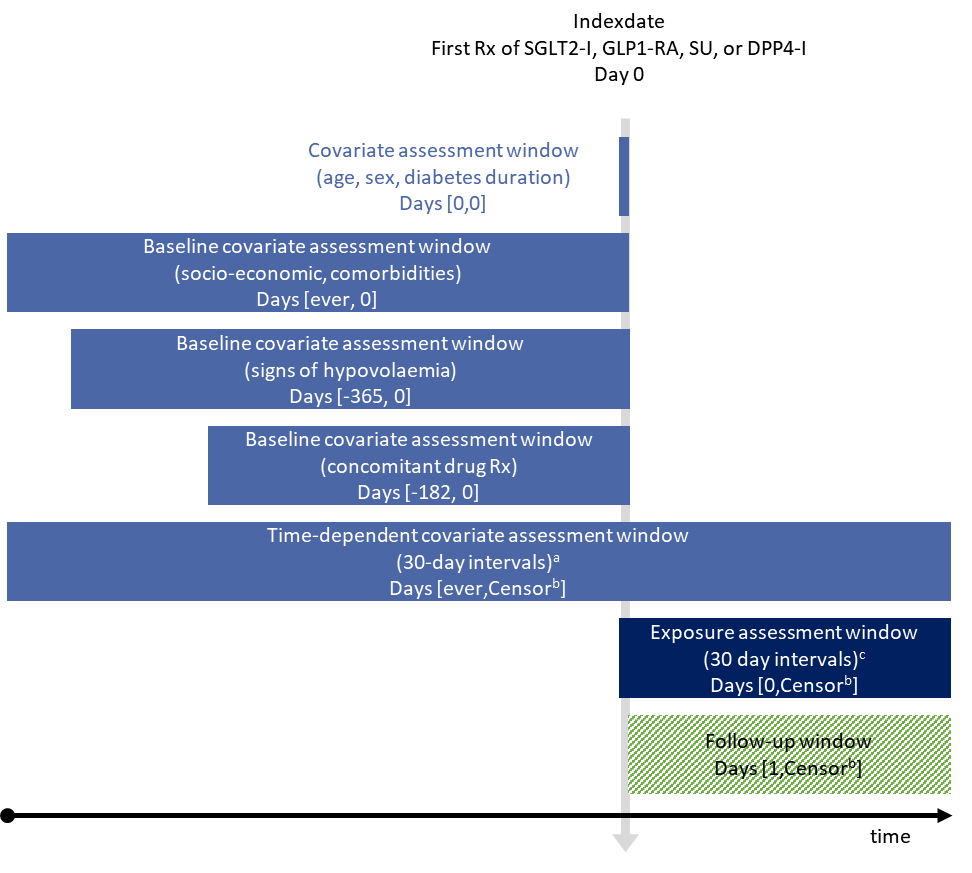


Abbreviations: Rx = prescription, SGLT2-I = sodium-glucose co-transporter-2 inhibitor, GLP1-RA = glucagon-like peptide-1 receptor agonist, SU = sulfonylurea, DPP4-I = dipeptidyl peptidase-4 inhibitor, GLD = glucose lowering drug.

^a^ Look back period varied per type of covariate. From the beginning of each interval the following covariates were considered: age, sex, diabetes duration (at the start of interval); socio-economic variables (ever before); the presence of comorbidities (ever before); and the use of drugs (half a year before).

^b^ The first occurrence of end of data collection, emigration, death, or the outcome of interest.

^c^ An interval was labeled as current (1-90 days) use or past (>90 days) use based on the most recent non-insulin GLD prescription before the start of the interval.

**Table S1** Risk of LLA, forefoot amputation and DFU in current use of different NIGLDs compared to DPP4-I use (sensitivity analysis 1).

|  | **Number of Events** | **IR (/1000 PY** | **Age/sex adjusted HR (95%CI)** | **Fully adjusted HR SGLT2-I model ^b^ (95%CI)** | **Fully adjusted HR GLP1-RA model ^c^ (95%CI)** |
| --- | --- | --- | --- | --- | --- |
| **LLA (N=564)** |  |  |  |  |  |
| Current DPP4-I use | 149 | 2.18 | reference | reference | reference |
| Current SGLT2-I use | 36 | 2.30 | 1.23(0.85-1.78) | 1.23(0.85-1.79) | 1.23(0.85-1.80) |
| Current GLP1-RA use | 55 | 1.51 | 0.87(0.64-1.19) | 0.65(0.47-0.89) | 0.64(0.47-0.88) |
| Current SU use | 54 | 2.14 | 0.99(0.72-1.35) | 1.12(0.82-1.54) | 1.13(0.82-1.55) |
| Current combined use ^a^ | 90 | 2.48 | 1.26(0.97-1.64) | 1.12(0.85-1.46) | 1.13(0.87-1.49) |
| Current other NIGLD use | 59 | 1.87 | 0.91(0.67-1.23) | 0.86(0.63-1.17) | 0.91(0.67-1.23) |
|  |  |  |  |  |  |
| **Forefoot amputation (N=367)** |  |  |  |  |  |
| Current DPP4-I use | 97 | 1.42 | reference | reference | reference |
| Current SGLT2-I use | 22 | 1.41 | 1.05(0.66-1.68) | 0.98(0.61-1.58) | 0.97(0.60-1.57) |
| Current GLP1-RA use | 40 | 1.10 | 0.91(0.62-1.31) | 0.63(0.43-0.93) | 0.63(0.43-0.92) |
| Current SU use | 34 | 1.34 | 0.96(0.65-1.43) | 1.09(0.74-1.62) | 1.11(0.74-1.65) |
| Current combined use ^a^ | 64 | 1.76 | 1.30(0.95-1.79) | 1.06(0.76-1.46) | 1.09(0.79-1.51) |
| Current other NIGLD use | 42 | 1.33 | 0.97(0.67-1.39) | 0.87(0.61-1.26) | 0.90(0.63-1.30) |
|  |  |  |  |  |  |
| **DFU (N=1,524)** |  |  |  |  |  |
| Current DPP4-I use | 392 | 5.78 | reference | reference | reference |
| Current SGLT2-I use | 101 | 6.51 | 1.18(0.95-1.47) | 1.06(0.85-1.33) | 1.09(0.87-1.37) |
| Current GLP1-RA use | 240 | 6.69 | 1.29(1.09-1.51) | 1.01(0.85-1.19) | 1.00(0.84-1.17) |
| Current SU use | 125 | 4.97 | 0.87(0.71-1.06) | 0.90(0.74-1.10) | 0.92(0.75-1.12) |
| Current combined use ^a^ | 254 | 7.07 | 1.27(1.08-1.48) | 1.05(0.89-1.23) | 1.09(0.93-1.28) |
| Current other NIGLD use | 174 | 5.57 | 0.99(0.83-1.18) | 0.88(0.73-1.05) | 0.89(0.75-1.07) |

^a^ combined use of at least two of the following NIGLDs: SGLT2-I and/or GLP1-RA and/or SU and/or DPP4-I.

^b^ adjusted for age; sex; diabetes duration; income category; history of diabetic foot ulcer, neuropathy, atherosclerosis, osteomyelitis, retinopathy, hypertension, heart failure, ischaemic heart disease, or peripheral arterial disease; and the use of antithrombotic agents, lipid lowering drugs, potassium sparing diuretics, beta blockers, or angiotensin receptor blockers in the six months before the start of the exposure interval.

^c^ adjusted for age; sex; diabetes duration; income category; immigrant status; education; history of diabetic foot ulcer, neuropathy, atherosclerosis, peripheral arterial disease, hypertension retinopathy, heart failure, hyperlipidaemia, ischaemic heart disease, osteomyelitis, renal disease, pulmonary heart disease, or bacterial foot infection; and the use of loop diuretics, antithrombotic agents, potassium sparing diuretics, lipid lowering drugs, angiotensin receptor blockers, digoxin, angiotensin converting enzyme inhibitors, or calcium channel blockers in the six months before the start of the exposure interval.

The models have also been corrected for past NIGLD use (not shown, 121 LLAs, 68 LLAdis, 238 DFUs)

**Table S2** Risk of LLA in current use of different NIGLDs compared to SU use excluding LLA history at baseline (sensitivity analysis 2).

|  | **Number of LLAs (N=470)** | **IR (/1000 PY** | **Age/sex adjusted HR (95%CI)** | **Fully adjusted HR SGLT2-I model ^b^ (95%CI)** | **Fully adjusted HR GLP1-RA model ^c^ (95%CI)** |
| --- | --- | --- | --- | --- | --- |
|  |  |  |  |  |  |
| Current DPP4-I use | 113 | 1.66 | 0.87(0.62-1.23) | 0.79(0.56-1.12) | 0.80(0.57-1.13) |
| Current SGLT2-I use | 29 | 1.86 | 1.11(0.69-1.78) | 1.05(0.65-1.69) | 1.05(0.65-1.69) |
| Current GLP1-RA use | 43 | 1.19 | 0.79(0.52-1.19) | 0.53(0.35-0.81) | 0.51(0.33-0.78) |
| Current SU use | 47 | 1.87 | reference | reference | reference |
| Current combined use ^a^ | 78 | 2.16 | 1.25(0.87-1.80) | 0.97(0.67-1.41) | 0.96(0.66-1.40) |
| Current other NIGLD use | 53 | 1.69 | 0.93(0.63-1.38) | 0.75(0.51-1.12) | 0.77(0.52-1.15) |

^a^ combined use of at least two of the following NIGLDs: SGLT2-I and/or GLP1-RA and/or SU and/or DPP4-I.

^b^ adjusted for age; sex; diabetes duration; income category; history of diabetic foot ulcer, neuropathy, atherosclerosis, osteomyelitis, retinopathy, hypertension, heart failure, ischaemic heart disease, or peripheral arterial disease; and the use of antithrombotic agents, lipid lowering drugs, potassium sparing diuretics, beta blockers, or angiotensin receptor blockers in the six months before the start of the exposure interval.

^c^ adjusted for age; sex; diabetes duration; income category; immigrant status; education; history of diabetic foot ulcer, neuropathy, atherosclerosis, peripheral arterial disease, hypertension retinopathy, heart failure, hyperlipidaemia, ischaemic heart disease, osteomyelitis, renal disease, pulmonary heart disease, or bacterial foot infection; and the use of loop diuretics, antithrombotic agents, potassium sparing diuretics, lipid lowering drugs, angiotensin receptor blockers, digoxin, angiotensin converting enzyme inhibitors, or calcium channel blockers in the six months before the start of the exposure interval.

The models have also been corrected for past NIGLD use (not shown, 107 LLAs)
